# Accuracy of MRI diagnosis of early osteonecrosis of the femoral head: a meta-analysis and systematic review

**DOI:** 10.1186/s13018-018-0836-8

**Published:** 2018-07-04

**Authors:** Ya-Zhou Zhang, Xu-Yang Cao, Xi-Cheng Li, Jia Chen, Yue-Yuan Zhao, Zhi Tian, Wang Zheng

**Affiliations:** Department of Orthopedics, Heibei General Hospital, No. 348 Heping East Road, Shijiazhuang, 050051 Hebei China

**Keywords:** Meta-analysis, Early osteonecrosis of the femoral head, Magnetic resonance imaging, Diagnostic accuracy

## Abstract

**Objective:**

To evaluate the overall diagnostic value related to magnetic resonance imaging (MRI) in patients with early osteonecrosis of the femoral head.

**Methods:**

By searching multiple databases and sources, including PubMed, Cochrane, and Embase database, by the index words updated in December 2017, qualified studies were identified and relevant literature sources were also searched. The qualified studies included prospective cohort studies and cross-sectional studies. Heterogeneity of the included studies were reviewed to select proper effect model for pooled weighted sensitivity, specificity, and diagnostic odds ratio (DOR). Summary receiver operating characteristic (SROC) analyses were performed for meniscal tears.

**Results:**

Forty-three studies related to diagnostic accuracy of MRI to detect early osteonecrosis of the femoral head were involved in the meta-analysis. The global sensitivity and specificity of MRI in early osteonecrosis of the femoral head were 93.0% (95% CI 92.0–94.0%) and 91.0% (95% CI 89.0%–93.0%), respectively. The global positive likelihood ratio and global negative likelihood ratio of MRI in early osteonecrosis of the femoral head were 2.74 (95% CI 1.98–3.79) and 0.18 (95% CI 0.14–0.23), respectively. The global DOR was 27.27 (95% CI 17.02–43.67), and the area under the SROC was 93.38% (95% CI 90.87%–95.89%).

**Conclusions:**

This review provides a systematic review and meta-analysis to evaluate the diagnostic accuracy of MRI in early osteonecrosis of the femoral head. Moderate to strong evidence indicated that MRI appears to be significantly associated with higher diagnostic accuracy for early osteonecrosis of the femoral head.

## Background

Avascular Necrosis of Femur Head (ANFH), or osteonecrosis of the femoral head, is a pathologic process, which was first seen in the weight-bearing area of the femur. The stress can lead to bone trabecular structure injury (microfracture) and influence the repair process of the femur, and if not managed timely, it leads to the collapse and deformation of the femur. With many etiological factors, ANFH results from interruption of blood supply to the bone and then leads to ischemic necrosis. ANFH can be divided in traumatic ANFH and non-traumatic ANFH with the non-traumatic ANFH further dividing into steroid-induced and alcoholic non-traumatic ANFH and so on. The timely treatment of early ANFH could promote the recovery the disease. However, in the late stage, it results in femur collapse, loss of hip function, and a very poor outcome that affects the quality of life. Therefore, the early diagnosis of ANFH is of great significance [1–3].

Several methods for early diagnosis of ANFH have been proposed, including MRI, SPECT, CT, X-ray, DSA, and laser Doppler with different characteristics. MRI has been characterized as being non-invasive, rapid and high sensitive, and commonly used by many clinicians [4–6]. Furthermore, MRI has been used in many studies in the diagnosis of early ANFH. Therefore, in this paper, a systematic review and meta-analysis of all qualified studies were performed to explore the diagnosis accuracy of MRI in early ANFH.

## Methods

### Search strategy

The following electronic databases were searched from their inception to December 2017: The Cochrane, PubMed, Embase database, for all the qualified trails that analyze the diagnostic accuracy of MRI of early osteonecrosis of the femoral head. Other related articles and reference materials were also identified for additional available studies. The literatures were searched independently by two investigators, and a third investigator was involved to reach an agreement.

### Study selection

The studies that met the following criteria were included in our review: (1) prospective cohort study or cross-sectional study; (2) the research objects are patients suspected with early osteonecrosis of the femoral head without other serious diseases; (3) the studies provided the data of true positive (TP), false positive (FP), false negative (FN), and true negative (TN); and (4) the publications were only available in English and Chinese.

The studies that met the following criteria were excluded in our review: (1) repeat publications, or shared content and results; (2) case report, theoretical research, conference report, systematic review, meta-analysis, expert comment, and economic analysis; (3) the outcomes were not relevant; and (4) two or more results of the TP, FP, FN, and TN were zero.

### Data extraction and quality assessment

Two independent investigators extracted the following data based on predefined criteria. Differences were settled by discussion with a third reviewer. The analyses data were extracted from all the included studies and consisted of two parts: basic information and main outcomes. The first part was about the basic information: the author name, the sample size, the percentage of male, and the age. The second part was the clinical outcomes. A 2 × 2 contingency table was constructed for each selected study; the results corresponding to the gold standard and MRI were selected as positive or negative. The data included true positive (TP), false positive (FP), false negative (FN), and true negative (TN). In studies in which one single cell in the 2 × 2 contingency table had a value of 0, 0.5 were added to all of the cells for calculation. Sensitivity, specificity, and likelihood ratio were calculated respectively, and the diagnostic odds ratio (DOR) was used as the measure of diagnostic accuracy. A DOR value of 1 indicates a test without discriminatory power, and the higher the DOR value is, the greater the degree of relevance of the assessed diagnostic test. The studies were performed by two reviewers independently. Any arising difference was resolved by discussion.

### Statistical analysis

All statistical analyses were performed in the STATA 10.0 (TX, USA). Chi-squared and *I*^2^ tests were used to assess the heterogeneity of clinical trial results and determine the analysis model (fixed-effects model or random-effects model). When the chi-squared test *P* value was ≤ 0.05 and *I*^2^ test value was > 50%, it was defined as high heterogeneity and assessed by random-effects model. When the chi-squared test *P* value was > 0.05 and *I*^2^ tests value was ≤ 50%, it was defined as acceptable heterogeneity data and assessed by fixed-effects model. For further assessment of heterogeneity, diagnostic threshold analysis was performed based on the correlation (Spearman’s) between the logit of sensitivity and the logit of [1-specificity]. When a threshold effect occurs, the sensitivity and specificity of the investigated study exhibits negative correlation (or a positive correlation between sensitivity and [1-specificity]). Therefore, a strong positive correlation between sensitivity and [1-specificity] suggests the presence of a threshold effect. When heterogeneity caused by threshold effect was observed, a summary receiver operating characteristic (SROC) curve was plotted. This method was appropriate given that the global sensitivity and specificity values were overestimated. In such cases, analysis of the ROC panel points, as well as analysis of the SROC curve, was recommended. Deeks’ Funnel Asymmetry Plot was used to identify the publication bias.

## Results

### Characteristics of included studies

**Fig. 1:**
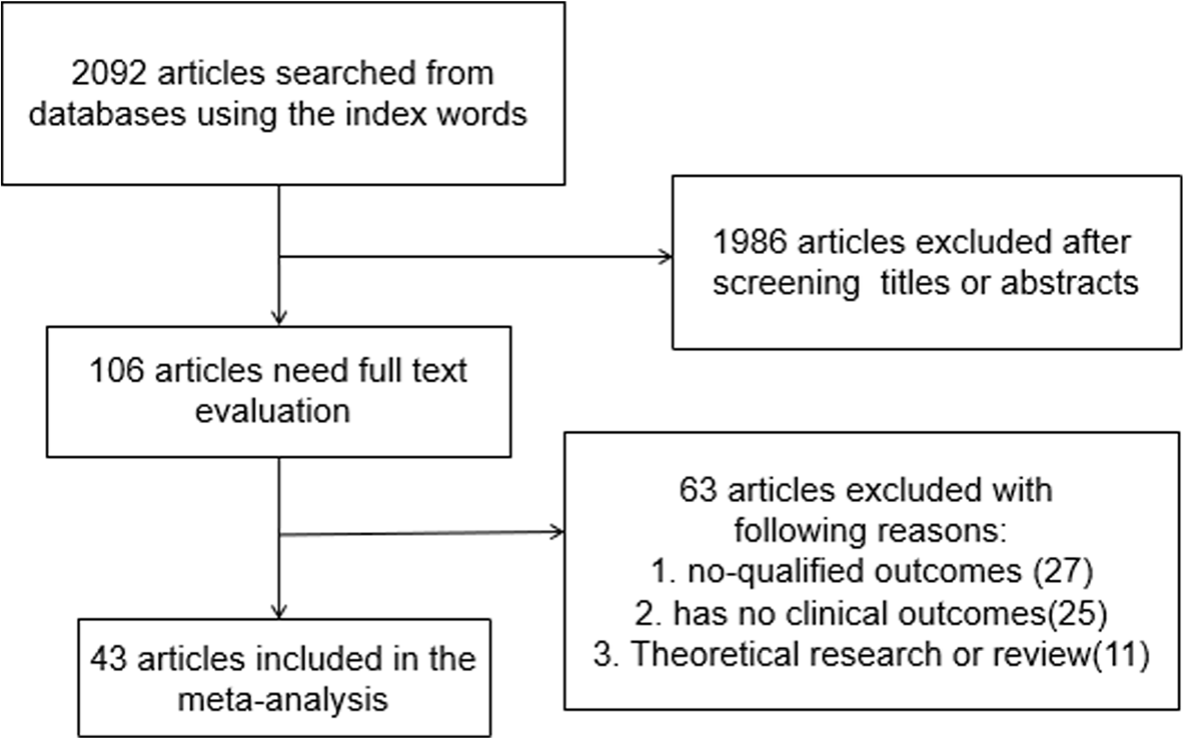
Flow diagram of the literature search and selection process

A total of 2092 articles were searched by the indexes. After screening the titles and abstracts, 1986 articles were excluded, leaving 106 articles for further selection. During full-text screening, 63 articles were excluded due to the following criteria: unqualified outcomes [7], theoretical research or review [8], and has non clinical outcome [9]. At last, 43 studies [7–50] with 3133 hips were involved in the final meta-analysis. The selection process was presented in Fig. 1. The main characteristics of the included studies were summarized in Table 1. The basic information included number of hips, age, and gender.

**Table 1:** The basic characteristics description of included studies

Study	No. of hip	Gender	Age
Genez BM 1998	11	4 M, 3F	11–35
Robinson HJ 1989	96	–	–
Hauzeur JP 1989	49	–	–
Zhang X 1994	30	26 M	24–58
Ryu JS 2002	32	14 M, 10 F	39.5
Liu Jihua 2004	72	40 M, 8 F	43.6
Chen Lei 2005	62	3 M, 21 F	31.8
Zhou Hongmei 2006	91	23 M, 18 F	30–60
Xie Zhongwei 2010	68	34 M, 16 F	34
Sun Lili 2015	50	36 M, 14 F	50.1
Fang Wu 2017	35	20 M, 15 F	57.1
Feng Zhanyou 2017	71	33 M, 38 F	61.5
Cheng Houpei 2016	65	40 M, 25 F	41.14
Qiu Pengdong 2012	59	31 M, 23 F	38.5
Zheng Liwen 2013	98	35 F, 21 M	38.2
Cui Baoli 2014	114	–	32.3
Xie Yan 2014	81	37 M, 32 F	31–62
Jia Hong 2017	40	25 M, 15 F	56.6
Zhang Kaixiang 2016	100	62 M, 38 F	42.3
Lin Chen 2017	42	–	52.87
Lu Chun 2016	52	28 M, 24 F	51.2
Ding Qinmei 2011	62	31 M, 15 F	41.2
Chen Longhua 2015	196	75 M, 45 F	58.4
Luo Zian 2017	93	39 M, 21 F	32.3
Tan Zhihong 2016	131	54 M, 32 F	58.68
Wang Yuli 2017	50	38 M, 12 F	53.8
Wang Wenbin 2012	72	29 M, 11 F	52.5
Liu Xianzhi 2017	29	16 M, 13 F	52.26
Liu Feng 2017	43	25 M, 18 F	58.14
Guo Hongbin 2017	70	39 M, 31 F	44.86
Lin Yi 2015	90	30 M, 20 F	45.6
Wang Linhong 2014	183	60 M, 40 F	47
Fang Chaohui 2014	122	56 M, 38 F	64.8
Li Yan 2014	98	69 M, 29 F	51.52
Liu Dailiang 2015	86	23 M, 20 F	55.36
Xiang Zhenghua 2014	61	35 M, 26 F	52.8
Wu Shiping 2017	75	51 M, 24 F	40.7
Wang Kun 2017	56	34 M, 22 F	38.74
Cai Huaiwei 2017	69	45 M, 24 F	54.6
Li Yanming 2015	37	–	52.6
Ji Xuewen 2017	50	28 M, 22 F	48.3
Wang Sihe 2013	86	32 M, 27 F	32.4
Shen Wen 2014	56	21 M, 15 F	45.3

### Diagnostic accuracy

All the included studies reported the results of the accuracy of MRI of early osteonecrosis of the femoral head. Based on the correlation (Spearman’s *R* = − 0.209, *P* = 0.589) between the logit of sensitivity and the logit of [1-specificity], there was no threshold effect.

**Fig. 2:**
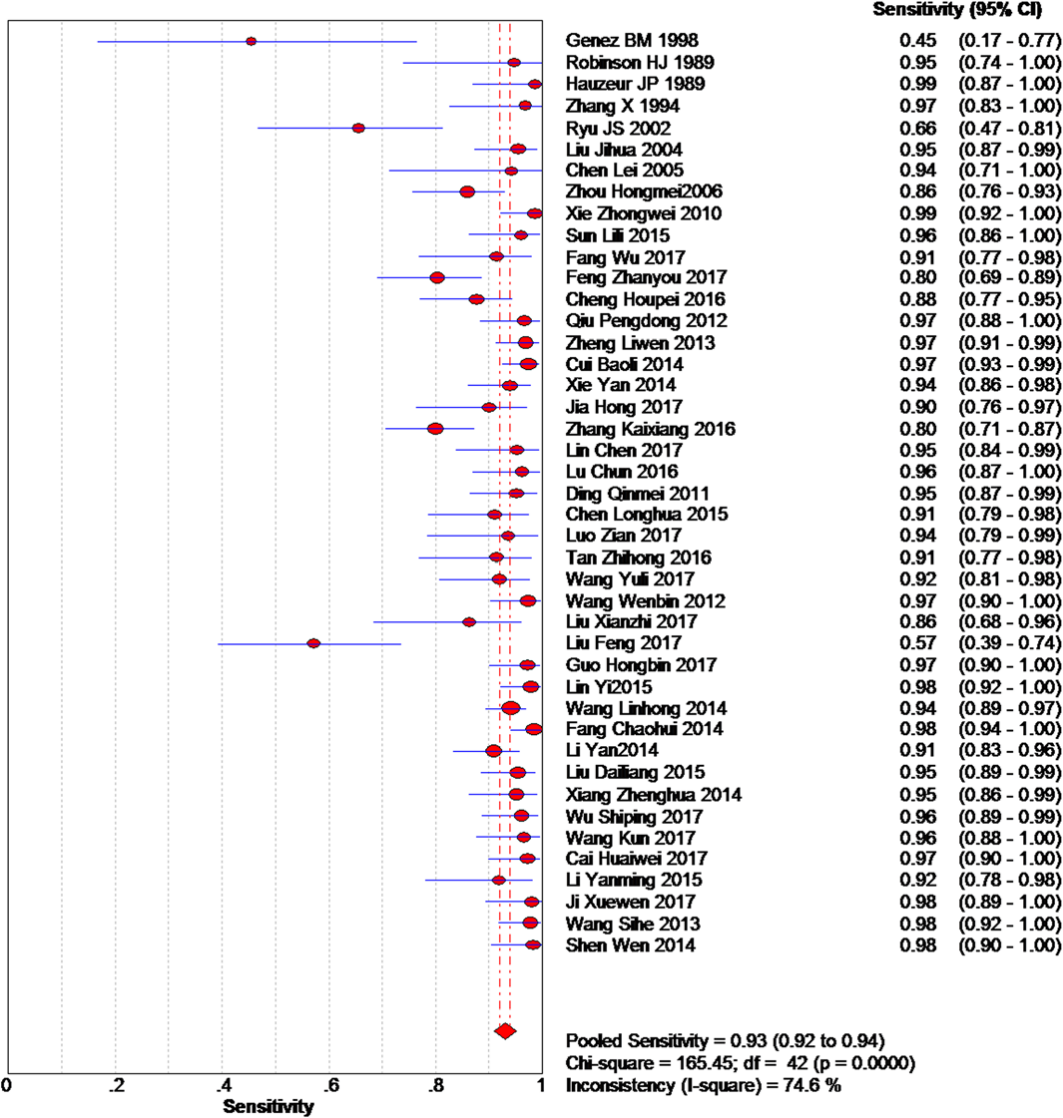
Forest plot showing the sensitivity values of MRI of early osteonecrosis of the femoral head

**Fig. 3:**
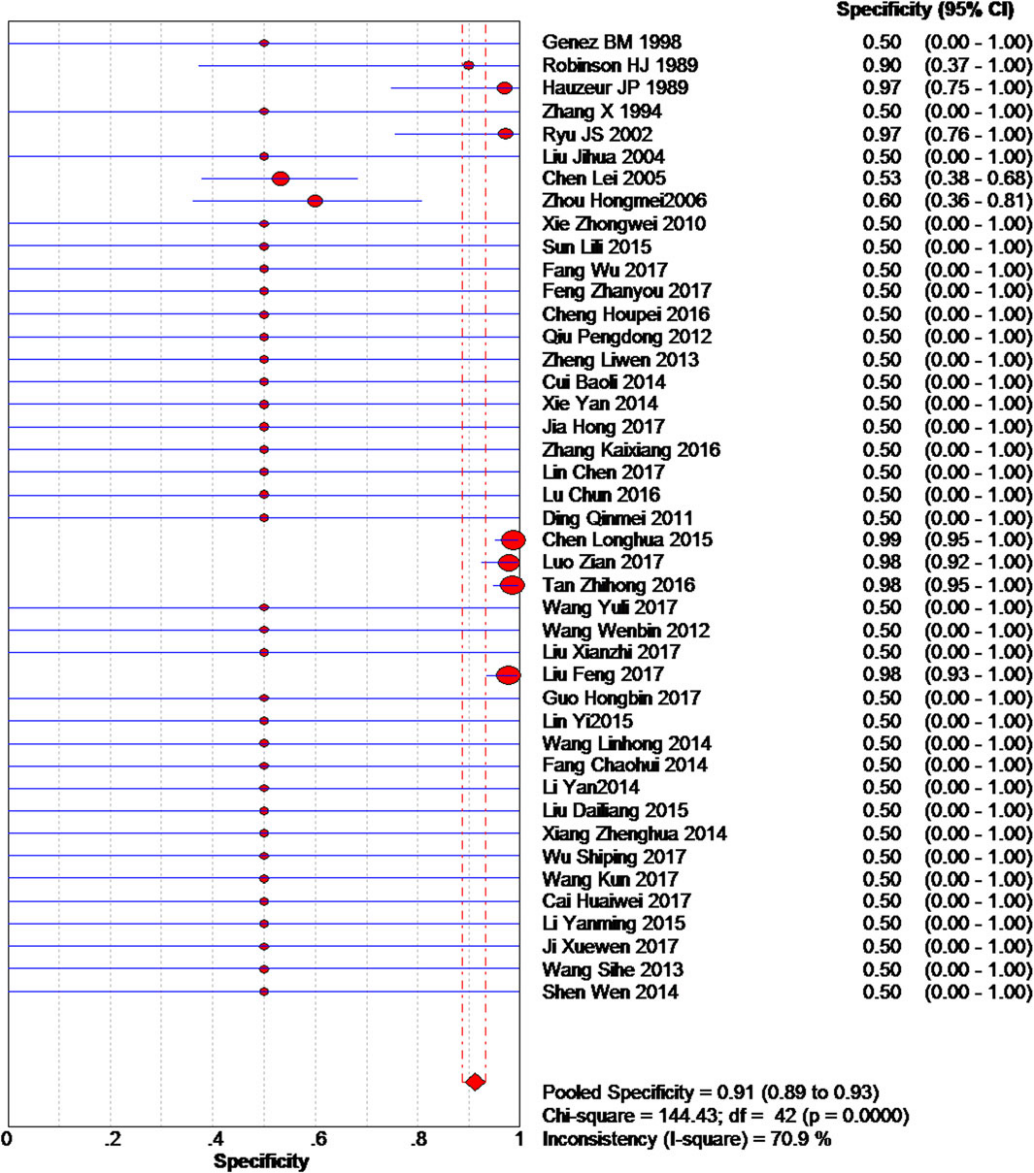
Forest plot showing the specificity values of MRI of early osteonecrosis of the femoral head

Based on the chi-squared test (*Q* = 166.45, *P* = 0.000) and *I*^2^ tests (*I*^2^ = 74.6%), heterogeneity was high, so we chose the random-effects model to analyze the sensitivity. The global sensitivity was 93.0% (95% CI 92.0–94.0%, Fig. 2). Based on the chi-squared test (*Q* = 144.43, *P* = 0.000) and *I*^2^ tests (*I*^2^ = 70.9%), heterogeneity was high. Therefore, we chose the random-effects model to analyze the specificity, and the global specificity was 91.0% (95%CI 89.0–93.0%, Fig. 3).

**Fig. 4:**
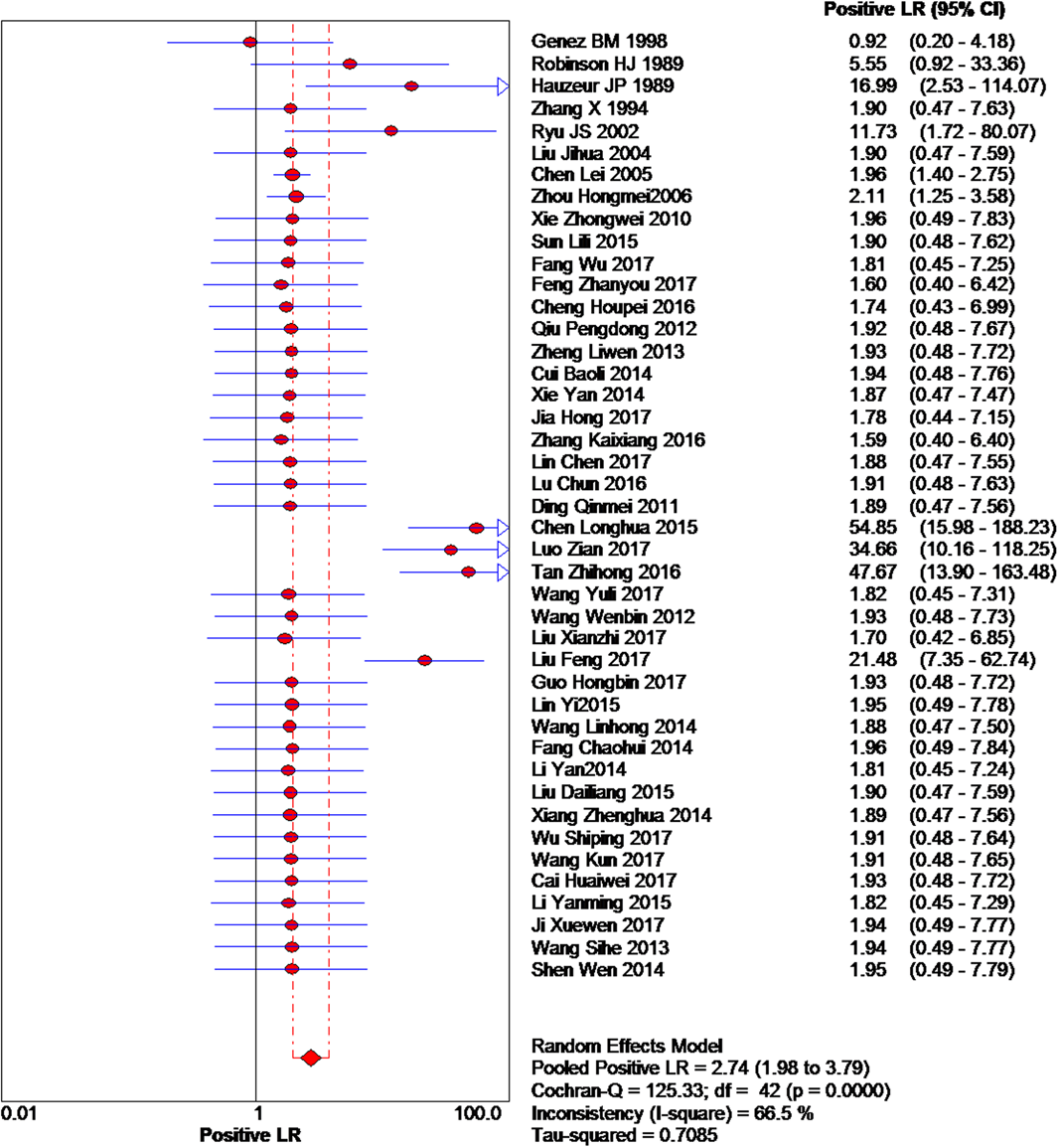
Forest plot showing the positive likelihood ratio of MRI

**Fig. 5:**
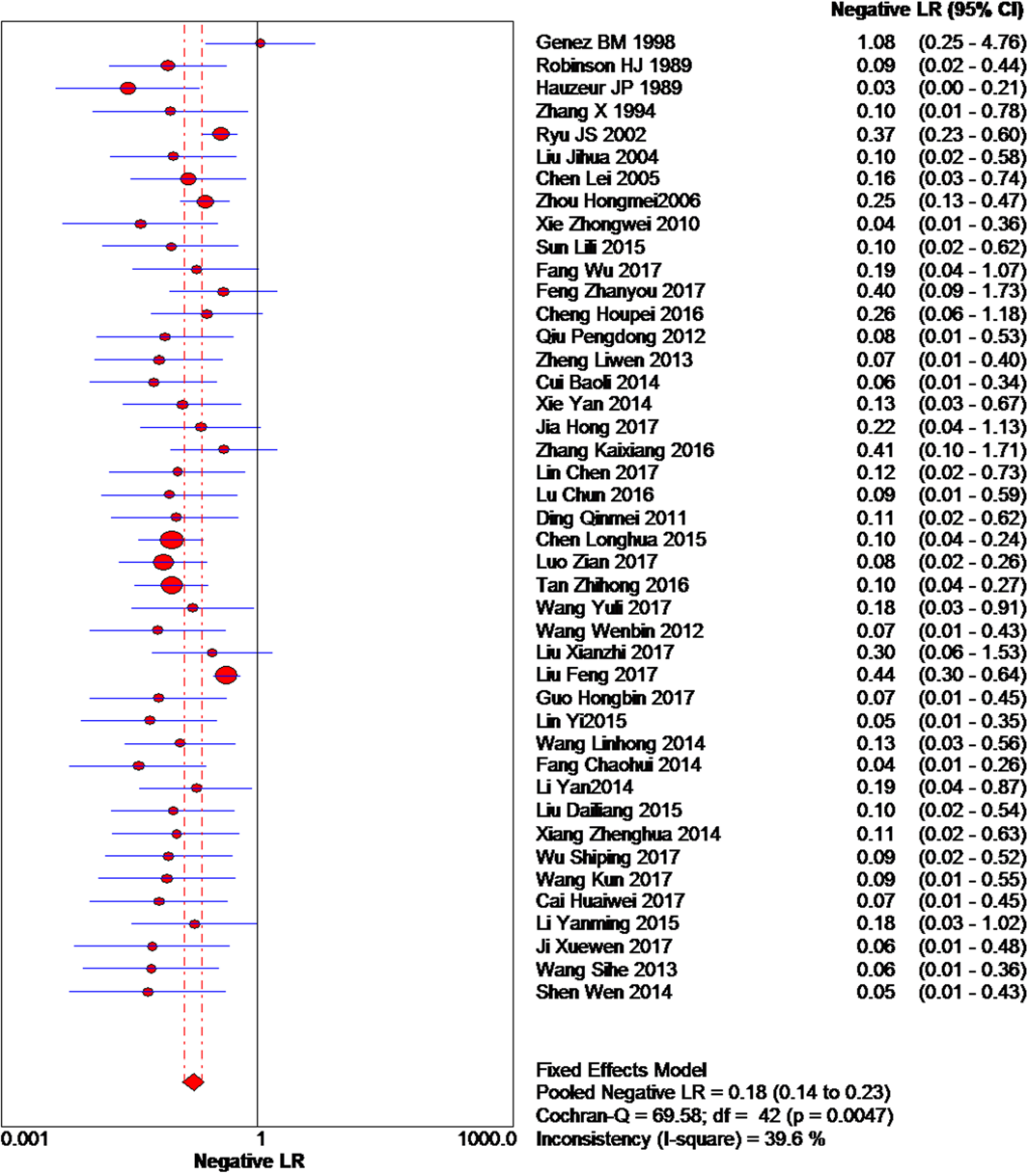
Forest plot showing the negative likelihood ratio of MRI

Based on the chi-squared test (*Q* = 125.33, *P* = 0.000) and *I*^2^ tests (*I*^2^ = 66.5%), heterogeneity was high, so we chose random-effects model to analyze the positive likelihood ratio, and the global positive likelihood ratio was 2.74 (95% CI 1.98–3.79, Fig. 4). Therefore, a positive MRI result was increased by 2.74-fold in the odds of an accurate diagnosis of patients who actually had early osteonecrosis of the femoral head. Based on the chi-squared test (*Q* = 69.58, *P* = 0.005) and *I*^2^ tests (*I*^2^ = 39.6%), with low heterogeneity, we chose the fixed-effects model to analyze the negative likelihood ration. The global negative likelihood ratio was 0.18 (95% CI 0.14–0.23, Fig. 5), indicating the use of MRI, which was close to zero. Specifically, the odds of a false-positive result were increased by only a factor of 0.18.

**Fig. 6:**
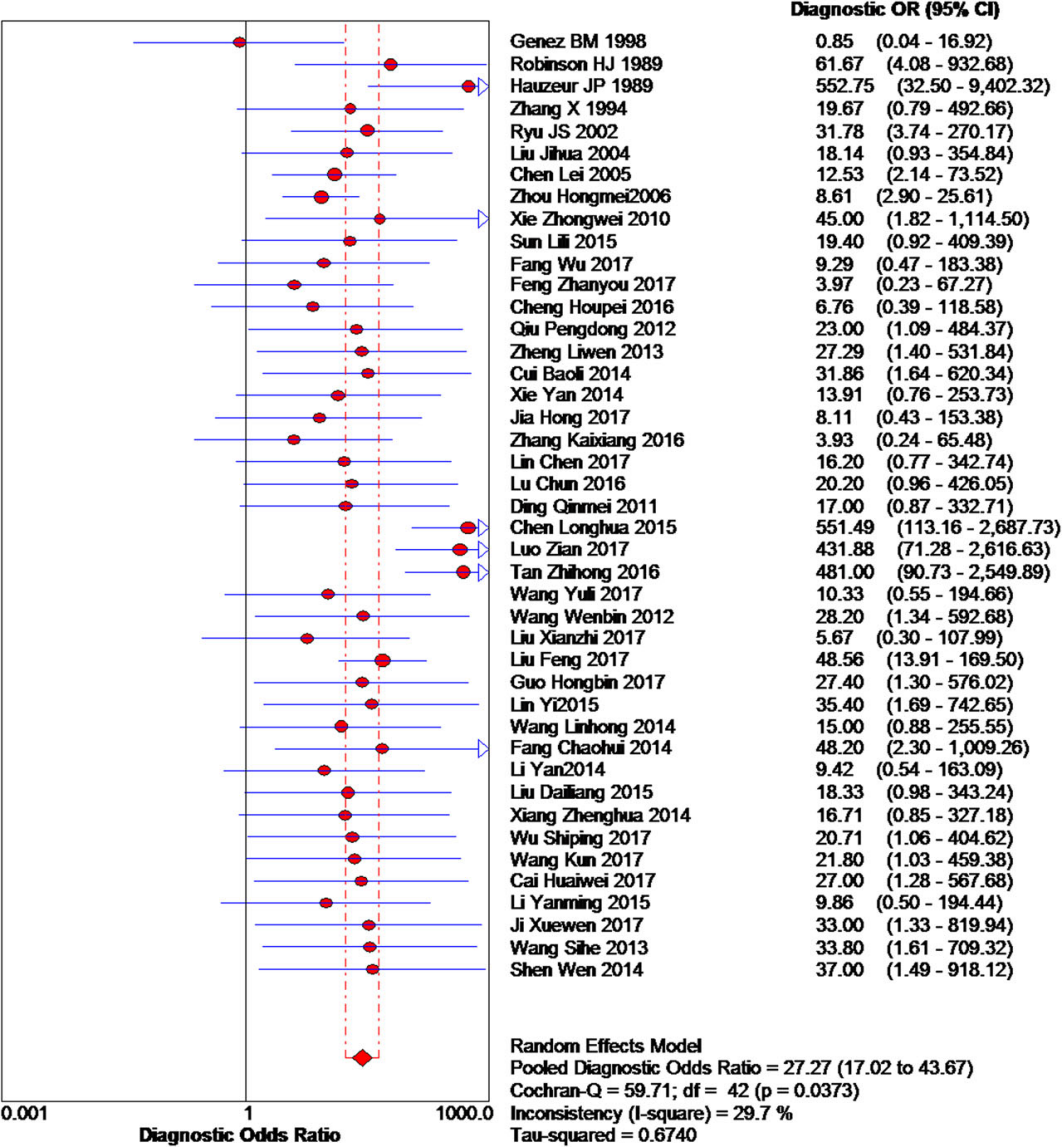
Forest plot showing the diagnostic odds ratio of MRI of early osteonecrosis of the femoral head

**Fig. 7:**
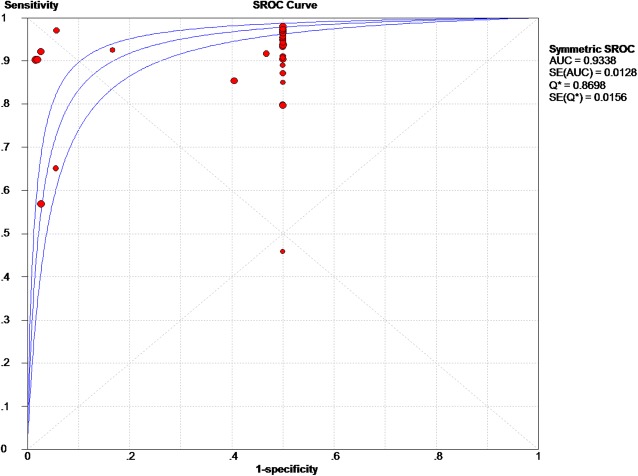
Summary ROC plots for diagnostic accuracy of MRI of early osteonecrosis of the femoral head

Based on the chi-squared test (*Q* = 59.71, *P* = 0.037) and *I*^2^ tests (*I*^2^ = 29.7%), heterogeneity was low, so we chose the fixed-effects model to analyze the DOR, with the global DOR being 27.27 (95% CI 17.02–43.67, Fig. 6). And the odds of a positive MRI result were 27.27-fold higher among individuals with early osteonecrosis of the femoral head compared to those without the disease. The area under the SROC was 93.38% (AUC = 93.38%; 95% CI 90.87%–95.89%, Fig. 7), indicating high accuracy.

## Conclusions

Several systematic reviews and meta-analysis have been published concerning the diagnostic accuracy of MRI of early osteonecrosis of the femoral head. Li et al. [51] found that the sensitivity and specificity of MRI were 95%(95% CI 94–96%) and 77%(95% CI 70–83%), respectively. Moreover, the DOR was 31.89%(95% CI 17.32–58.70%), and the AUC under the SROC was 0.9166. MRI was associated with high diagnostic accuracy in the patients with suspected early ANFH. Song et al. [52], who included 21 articles, reported that MRI was more effective than CT in diagnosing ANFH. Significant statistical difference was identified between them (OR, 0.13; 95% CI 0.03–0.51). Su et al. [53], who included 8 studies of 515 patients, found the ANFH positive rate between CT and MRI was statistically significant (OR, 0.12; 95% CI 0.04–0.33), so as the early stage positive rate (OR, 0.45; 95% CI 0.26–0.78). Therefore, MRI appears to be a promising diagnostic tool for avascular necrosis of the femoral head.

However, there were several limitations in this analysis: (1) differences in the inclusion and exclusion criteria for patients, (2) different patients with previous disease and treatments were unavailable, (3) all the included studies were from English and Chinese articles, which may be the source of bias, (4) the fluency of technicians between different studies varied, and (5) pooled data were used for analysis, and individual patients’ data were unavailable, which limited a more comprehensive analysis.

In summary, in this systematic review and meta-analysis, MRI as a diagnostic method is associated with higher accuracy for detecting ANFH. More studies and randomized controlled trails with high-quality and large samples are warranted for further evaluation.

## Abbreviations

ANFH: Avascular Necrosis of Femur Head
FN: False negative
FP: False positive
MRI: Magnetic resonance imaging
SROC: Summary receiver operating characteristic
TN: True negative
TP: True positive

## References

[1] Mankin HJ (1992). Nontraumatic necrosis of bone (osteonecrosis). N Engl J Med.

[2] Castro FP, Barrack RL (2000). Core decompression and conservative treatment for avascular necrosis of the femoral head: a meta-analysis. Am J Orthop.

[3] Hong YC, Zhong HM, Lin T, Shi JB (2015). Comparison of core decompression and conservative treatment for avascular necrosis of femoral head at early stage: a meta-analysis. Int J Clin Exp Med.

[4] Nakamura T, Matsumoto T, Nishino M, Tomita K, Kadoya M (1997). Early magnetic resonance imaging and histologic findings in a model of femoral head necrosis. Clin Orthop Relat Res.

[5] Ficat RP (1985). Idiopathic bone necrosis of the femoral head. Early diagnosis and treatment. J Bone Joint Surg Br Vol.

[6] Jawad MU, Haleem AA, Scully SP (2012). In brief: Ficat classification: avascular necrosis of the femoral head. Clin Orthop Relat Res.

[7] Li YM (2015). Comparative analysis of CT and MRI in diagnosis of femoral head necrosis. World Latest Med Inform.

[8] Sun LL (2015). Comparative study of CT and MRI in diagnosis of 100 cases of femoral head necrosis. China Health Stand Manag.

[9] Wang YL (2017). The value of contrast MRI and CT in the early diagnosis of femoral head necrosis. Chin J Trauma Disabil Med.

[10] Zheng LW, Chen RH, Chen YH (2013). The application value of CT and MRI in early diagnosis of osteonecrosis of the femoral head. China Mod Doct.

[11] Cui BG (2014). The application value of CT and MRI in early diagnosis of osteonecrosis of the femoral head. Mod Med Imageol.

[12] Xie Y (2014). The value of CT and MRI in the diagnosis of early femoral head necrosis. Chin J Mod Drug Appl.

[13] Jia H, Xiao YX, Zhang J, An L (2017). The value of CT and MRI in the diagnosis of femoral head necrosis. Gansu Med J.

[14] Fang W, Yang WJ, Liang TH, Huang RS (2017). Application value of CT and MRI in the diagnosis of early femoral head necrosis. J Baotou Med Coll.

[15] Feng ZY, Ma Z, Qu JX, Chen W (2017). CT and magnetic resonance imaging (MRI) in the diagnosis of femoral head necrosis in comparison. Mod Diagn Treat.

[16] Zhang KX (2016). MRI and CT diagnosis of avascular necrosis of the femoral head. World Clin Med.

[17] Zi LL. Comparative analysis of CT and MRI in the diagnosis of early necrosis of femoral head. Mod Diagn Treat. 2015;(9):2093–4.

[18] Qiu PD (2012). Comparative analysis of CT and MRI in the diagnosis of early necrosis of femoral head. Modern Medical Imageology.

[19] Lin C, Ren CP (2017). Clinical value of MRI in the diagnosis of early necrosis of femoral head. J Med Imag.

[20] Sheng W (2014). Comparative analysis of diagnostic value of X - ray, CT and MRI in early diagnosis of avascular necrosis of femoral head. Mod Diagn Treat.

[21] Lu C (2016). Analysis on diagnosis value of MRI in early femoral head necrosis. Chin Foreign Med Res.

[22] Ding QM (2011). Comparison of X-ray, CT and MRI in the diagnosis of early avascular necrosis of femoral head. Chin J Primary Med Pharm.

[23] Wang SH (2013). Application of spiral CT and high field MRI in the early diagnosis of adult femoral head necrosis. Jiangxi Med J.

[24] Chen LH. Clinical value of CT and MRI in diagnosis of early osteonecrosis of femoral head in adults. Mod Inst Med Treat. 2015;(2):8–10.

[25] Luo ZA (2017). Clinical value of CT and MRI in diagnosis of early osteonecrosis of femoral head in adults. China Med Device Inform.

[26] Tan ZH, Wu Y, Zhang PM, Li XL (2016). Clinical value of CT and MRI in diagnosis of early osteonecrosis of femoral head in adults. Mod Med Imageol.

[27] Ji XW (2017). Patients with avascular necrosis of the femoral head using MRI, spiral CT examination contrast. China Reflexolocy.

[28] Cai HW (2017). Comparison of multislice spiral CT and MRI in the clinical diagnosis of femoral head necrosis. Int Med Health Guid News.

[29] Wang WB (2012). Comparative study of CT and MRI in patients with femoral head necrosis. Chin J CT MRI.

[30] Liu XZ, Mu CL (2017). Femoral head necrosis in patients with a comparative study of CT and mri diagnosis. J Imag Res Med Appl.

[31] Liu F (2017). Comparative analysis of CT and MRI radiological diagnosis of femoral head necrosis. Mod Diagn Treat.

[32] Guo HB (2017). Comparative analysis of CT and MRI in diagnosis of avascular necrosis of femoral head. Pract Clin J Integ Tradit Chin West Med.

[33] Lin Y (2015). Comparison of CT and magnetic resonance imaging in diagnosis and treatment of femoral head necrosis. Guide China Med.

[34] Wang LH (2014). Effect of CT and MRI in diagnosing femoral head necrosis. Contemp Med.

[35] Fang CH (2014). Diagnostic value of two methods of MRI and CT in avascular necrosis of femoral head. China Med Pharm.

[36] Li Y (2014). MRI combined with CT in the diagnosis of avascular necrosis of the femoral head. J Med Imaging.

[37] Zhang J, Peng WX, Y. YF (2016). Observation on the MRI diagnosis on ischemic bone necrosis and surgical treatment. Chin Commun Doct.

[38] Liu DL (2015). The value of CT and MRI examination in the early diagnosis of adult femoral head necrosis. Med Inf.

[39] Xiang ZH (2014). Low field strength MRI diagnosis of avascular necrosis of femoral head. China Foreign Med Treat.

[40] Wu SP (2017). Diagnostic value of multislice spiral CT and mill on femoral head necrosis. Henan Med Res.

[41] Wang K (2017). Evaluation of the value of multislice spiral CT and MRI in the diagnosis of femoral head necrosis. Diet Health.

[42] Xie ZW, Yuan YH (2010). Early diagnosis of avascular necrosis of femoral head in adults. Chin J Lab Diagn.

[43] Genez BM, Wilson MR, Houk RW, Weiland FL, Unger HR, Shields NN (1988). Early osteonecrosis of the femoral head: detection in high-risk patients with MR imaging. Radiology.

[44] Robinson HJ, Hartleben PD, Lund G, Schreiman J (1989). Evaluation of magnetic resonance imaging in the diagnosis of osteonecrosis of the femoral head. Accuracy compared with radiographs, core biopsy, and intraosseous pressure measurements. J Bone Joint Surg Am.

[45] Hauzeur JP, Pasteels JL, Schoutens A, Hinsenkamp M, Appelboom T, Chochrad I (1989). The diagnostic value of magnetic resonance imaging in non-traumatic osteonecrosis of the femoral head. J Bone Joint Surg Am.

[46] Zhang X, Hu CM, Zhao GK, Sun L, Qin DM (1994). The value of magnetic resonance imaging in the early diagnosis of noninvasive avascular necrosis of femoral head. Chin J Surg.

[47] Ryu JS, Kim JS, Moon DH, Kim SM, Shin MJ, Chang JS (2002). Bone SPECT is more sensitive than MRI in the detection of early osteonecrosis of the femoral head after renal transplantation. J Nuclear Med.

[48] Liu JH, Zhang SP, Wang SH, Zuo SY (2006). Comparative study of radionuclide scanning and MRI in diagnosis of avascular necrosis of femoral head in early adults. Acta Acad Med Qingdao Univ.

[49] Chen L, Hong N, Du XK (2005). Avascular necrosis in severe acute respiratory syndrome: MR imaging with radionuclide correlation. Chin J Med Imag Technol.

[50] Zhou HM, Li RG, Cui B, Tang FM, Huang BL (2006). Diagnostic value of CT and MRI in the early stage of adult femoral head necrosis. J Qiqihar Univ Med.

[51] Li YX, Jiang PQ (2013). Meta-analysis of MRI diagnosis of early avascular necrosis of femoral head. Chin Foreign Med Res.

[52] Song WT, Li Z, Li XM, Yang XF (2010). Meta-analysis of CT and MRI diagnosis of avascular necrosis of femoral head. J Pract Radiol.

[53] Su JJ, Wu GY, Zhu L, Liu GB (2011). Meta-analysis of CT and MRI diagnosis of avascular necrosis of femoral head. J New Med.

